# Developmental constraint underlies the replicated evolution of grass awns

**DOI:** 10.1111/nph.20268

**Published:** 2024-11-18

**Authors:** Erin Patterson, Dana R. MacGregor, Michelle M. Heeney, Joseph Gallagher, Devin O'Connor, Benedikt Nuesslein, Madelaine Elisabeth Bartlett

**Affiliations:** ^1^ Department of Biology University of Massachusetts 611 N. Pleasant St Amherst MA 01002 USA; ^2^ Ecology and Evolutionary Biology University of Connecticut Storrs CT 06268 USA; ^3^ Rothamsted Research, Protecting Crops and the Environment, West Common Harpenden Hertfordshire AL5 2QJ UK; ^4^ Cornell University Ithaca NY 14853 USA; ^5^ US Department of Agriculture Agricultural Research Service, Forage Seed and Cereal Research Unit National Forage Seed Production Research Center 3450 SW Campus Way Corvallis OR 97331 USA; ^6^ Sainsbury Laboratory Cambridge University Cambridge CB2 1LR UK; ^7^ Department of Biology Colorado State University 1878 Campus Delivery Fort Collins CO 80523 USA; ^8^ Huck Institute Pennsylvania State University State College PA 16802 USA

**Keywords:** ancestral developmental potential, convergent evolution, developmental constraint, flower development, grass evolution, leaf development, parallel evolution, plant evo‐devo

## Abstract

- Replicated trait evolution can provide insights into the mechanisms underlying the evolution of biodiversity. One example of replicated evolution is the awn, an organ elaboration in grass inflorescences.
- Awns are likely homologous to leaf blades. We hypothesized that awns have evolved repeatedly because a conserved leaf blade developmental program is continuously activated and suppressed over the course of evolution, leading to the repeated emergence and loss of awns. To evaluate predictions arising from our hypothesis, we used ancestral state estimations, comparative genetics, anatomy, and morphology to trace awn evolution.
- We discovered that awned lemmas that evolved independently share similarities in developmental trajectory. In addition, in two species with independently derived awns and differing awn morphologies (*Brachypodium distachyon* and *Alopecurus myosuroides*), we found that orthologs of the *YABBY* transcription factor gene *DROOPING LEAF* are required for awn initiation. Our analyses of awn development in *Brachypodium distachyon*, *Alopecurus myosuroides*, and *Holcus lanatus* also revealed that differences in the relative expansion of awned lemma compartments can explain diversity in awn morphology at maturity.
- Our results show that developmental conservation can underlie replicated evolution and can potentiate the evolution of morphological diversity.

## Introduction

Phenotypic variation creates opportunities for natural selection to act. Conserved developmental pathways can shape the character of this phenotypic variation. This shaping is usually viewed as a negative constraint, where developmental conservation limits phenotypic variation (Prusinkiewicz *et al*., 2007; Wessinger & Hileman, 2016; Blount *et al*., 2018). However, there are cases where development acts to generate or potentiate phenotypic variation (Gould, 2002; West‐Eberhard, 2003; Rajakumar *et al*., 2012; Leichty & Sinha, 2021). For example, ants in the genus *Pheidole* share ancestral developmental potential to generate a supersoldier caste. Supersoldier ants appear naturally in two species, but can be induced by hormones in three additional species, indicating a conserved developmental pathway with the potential to facilitate phenotypic change (Rajakumar *et al*., 2012). Here, we show that developmental conservation in the grasses can act to potentiate (rather than limit) morphological diversity. We hypothesize that developmental conservation underlies the replicated evolution of a morphological trait (James *et al*., 2023) – the awned grass lemma.

**Fig. 1:**
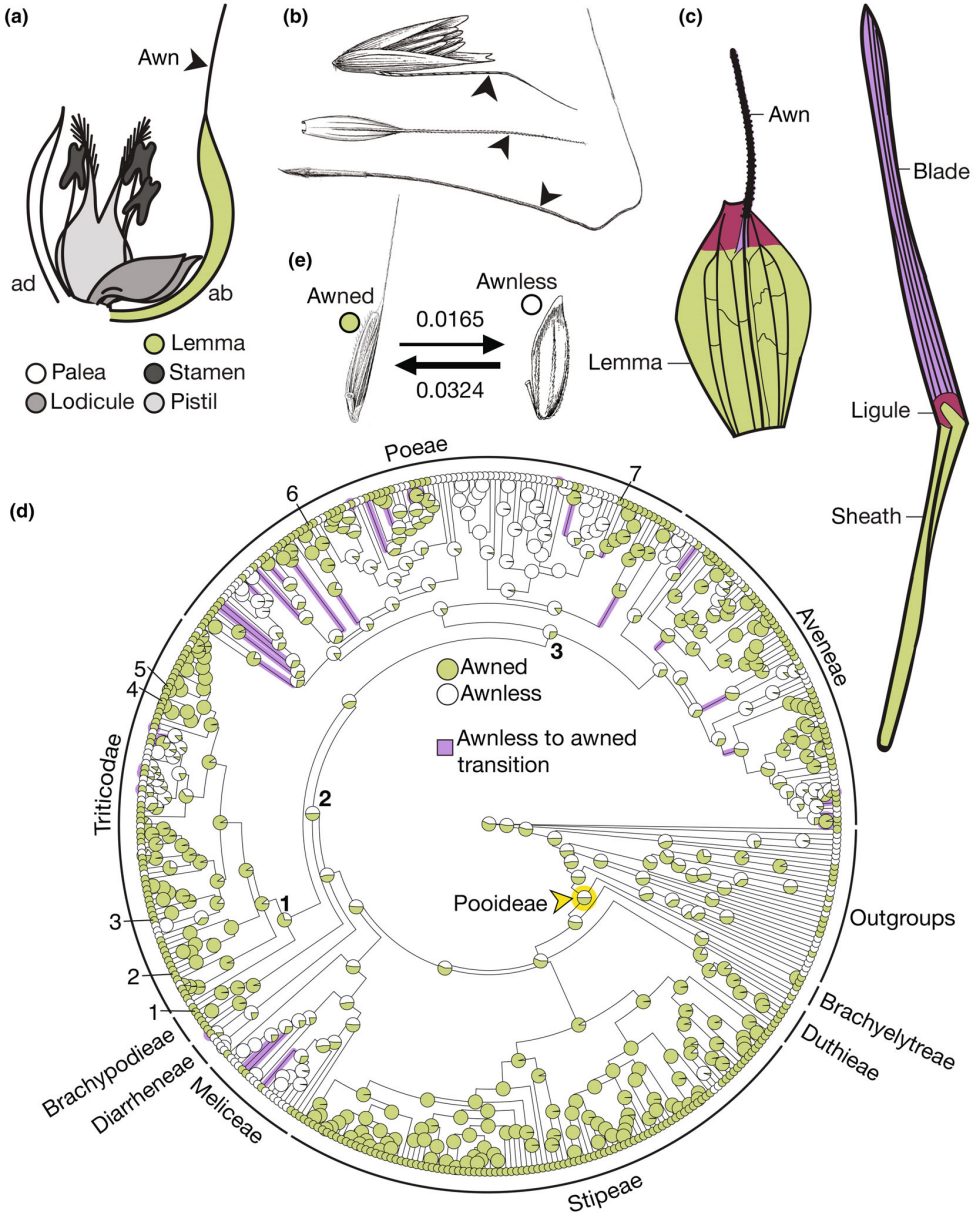
Awns have been gained, lost, and regained many times in the Pooideae. (a) Generalized grass flower diagram, awn noted with arrow. (b) Diversity of awns in the Pooideae. From top: abaxially insterted twisted geniculate awn (*Calamagrostis* sp.), apically inserted straight awn (*Secale cereale*), and apically inserted twisted geniculate awn (*Stipa spartea*). (c) Predicted homology of awned lemmas to leaves. (d) Ancestral state reconstruction for awn presence. Species of interest noted: (1) brachypodium (*Brachypodium distachyon*), (2) *Bromus tectorum*, (3) barley (*Hordeum vulgare*), (4) rye (*Secale cereale*), (5) wheat (*Triticum aestivum*), (6) velvetgrass (*Holcus lanatus*), (7) blackgrass (*Alopecurus myosuroides*). Nodes of interest noted: most recent common ancestor of (1) Triticodae, (2) Brachypodium and Triticodae, (3) Poeae and Aveneae. Purple branches indicate gain of awns. Yellow node indicates Pooideae most recent common ancestor. (e) Transition rates between awned and awnless states in (d). Lemma and leaf in (c) from Thi‐Tuyet‐Hoa (1965), redrawn with permission, copyright Meise Botanic Garden. Images in (b), and (e) from Hitchcock‐Chase Collection of Grass Drawings.

Lemmas are bract‐like organs that subtend grass flowers (Sajo *et al*., 2008; Kellogg, 2015; Patterson *et al*., 2023). Many lemmas bear bristle‐like extensions called awns (Fig. 1a,b). Awned lemmas have evolved multiple times, and lemma morphology and function varies extensively across the grasses (Linder *et al*., 2018; McAllister *et al*., 2018; Cavanagh *et al*., 2020; Petersen & Kellogg, 2022). Awns can also occur on other grass inflorescence organs: both paleas (likely sepal homologs), and glumes (bracts) can bear awns (Kellogg, 2015; Petersen & Kellogg, 2022). Many awns are short and simple, with no hypothesized functions. However, longer and more complex awns can contribute to grass grain development, dispersal, and seedling establishment (Yanez *et al*., 2018; Ntakirutimana & Xie, 2020; Cavanagh *et al*., 2020; Sanchez‐Bragado *et al*., 2023) features that have likely contributed to the grass family's extraordinary ecological success and dominance (Linder *et al*., 2018).

Awn function is tied to awn form. For example, lemmas with barbed awns may function in animal dispersal (Elbaum *et al*., 2007; Hua *et al*., 2015), while feathery awns may function in wind dispersal (Hensen & Müller, 1997; Yanez *et al*., 2018). Twisted geniculate awns, which have a proximal twisted column and knee‐like bend, are often hygroscopic and can twist and untwist to move grass grains across a surface (Peart, 1979; Raju, 2011). This movement may increase grain dispersal distance and help with seedling establishment by burying grains deeper underground (Garnier & Dajoz, 2001; Cavanagh *et al*., 2020; Morris, 2021). Awns in barley and wheat photosynthesize, and contribute photosynthate to developing grains (Abebe *et al*., 2009; Sanchez‐Bragado *et al*., 2023). Therefore, awned lines of wheat and barley have fewer, heavier grains than un‐awned lines (Weyhrich *et al*., 1994; Motzo & Giunta, 2002; Sanchez‐Bragado *et al*., 2023). Extra provisions in these heavier grains may help with seedling establishment (Nik *et al*., 2011; Linder *et al*., 2018; Muhsin *et al*., 2021; Petersen & Kellogg, 2022). Awns have also been implicated in preventing herbivory (Schöning *et al*., 2004; Ceradini & Chalfoun, 2017; Titulaer *et al*., 2018), as well as in modifying canopy temperature and grain shattering (Ntakirutimana & Xie, 2020). Thus, awned lemma form and function have diverged extensively.

Despite morphological and functional variation, all lemmas are leaf homologs (Thi‐Tuyet‐Hoa, 1965; Von Goethe & Miller, 2009; Patterson *et al*., 2023). Grass leaves contain three compartments along the proximal‐distal axis – the sheath, the ligule, and the blade (Lewis & Hake, 2016). In awned lemmas, awns are likely homologous to the blade compartment (Duval‐Jouve, 1871; Thi‐Tuyet‐Hoa, 1965; Fig. 1c). Therefore, awnless lemmas are predicted to have lost blade compartments.

Morphological trait loss is often associated with parallel genetic loss. However, genetic loss is unlikely to be the case in awn evolution. Gene loss accompanied the evolution of eyelessness in cavefish (Sifuentes‐Romero *et al*., 2020), of rootlessness in aquatic plants (Hepler *et al*., 2020; Ware *et al*., 2023), and of fungal cells that cannot crawl (Torruella *et al*., 2015; Prostak *et al*., 2021). In contrast to these cases, awn loss is easily reversible: awns have been gained, lost, and regained many times over the course of grass evolution (Humphreys *et al*., 2011; Petersen & Kellogg, 2022). In addition, many grass species have awnless lemmas, but vegetative leaves with a typical leaf blade, including *Zea mays* (corn), and many *Oryza sativa* (rice), *Sorghum bicolor* (sorghum), and *Triticum aestivum* (wheat) cultivars (Kellogg, 2015). These data indicate that even when awns are not present, the genes that regulate leaf blade (and awn) development are maintained in grass genomes.

The retention of a blade developmental program in awnless species may provide a cache of genetic material that, under the right circumstances, could be activated to generate phenotypic variation upon which selection could act. We hypothesize that awns are gained and lost through the repeated activation and suppression of a leaf blade developmental program in lemmas. Under this hypothesis, developmental constraint would not limit phenotypic variation, but instead potentiate its emergence (Gould, 2002; West‐Eberhard, 2003).

To test this hypothesis, we dissected awn evolutionary history in the grasses, focusing on the Pooideae subfamily, which includes the genetic experimental system *Brachypodium distachyon* (brachypodium), as well as many crops like barley, oats, and wheat (Schubert *et al*., 2019; Zhang *et al*., 2022). We evaluated two predictions arising from our hypothesis. Specifically, if a conserved developmental program underlies the replicated evolution of awns, we predicted that independently derived awns and leaves would have (1) conserved developmental trajectories and (2) conserved genes regulating their development. Our comparative analyses of anatomy, morphology, development, and genetics revealed the conserved developmental mechanisms underlying both the replicated evolution of awns, and the evolution of awn diversity.

## Materials and Methods

### Ancestral state estimation

We collected awn traits from GrassBase for species present in a time‐calibrated phylogeny (Schubert *et al*., 2019, Clayton *et al*., 2006), and mapped the traits to the tree. We estimated ancestral states for each trait and rates of transition between traits using a maximum likelihood approach from corHMM (Boyko & Beaulieu, 2021). For each categorical trait, we compared two Markov models: Equal Rates (ER), which holds all transition rates between traits to be equal, and All Rates Different (ARD), where all transition rates may freely differ. In all cases, the ARD model was preferred (with a smaller AICc value), and was used with stochastic character mapping to plot the likely state at each ancestral node (Revell, 2012). Briefly, once the model has been generated, stochastic mapping uses a Bayesian approach to repeatedly generate random histories that allow the tree to arrive at the extant tip states (Bollback, 2006). These repeated simulated histories were combined and the proportion of each state at each node was plotted. For phylogenetic signal of the binary presence trait, the *D*‐statistic was calculated using the R package caper. A *D* value of 1 indicates a trait is distributed randomly across a phylogeny, whereas a *D* value of 0 indicates strong phylogenetic signal, consistent with a trait evolving under Brownian motion (Fritz & Purvis, 2010; Orme, 2013).

### Plant materials and growth conditions

Brachypodium (*Brachypodium distachyon* (L.) P. Beauv.) *awnless1* mutant grains were developed by fast neutron mutagenesis in the Bd21 background (Derbyshire & Byrne, 2013). Genotyping of *awl1* was performed via multiplex PCR with primers spanning the deletion and a pair specific to a gene inside the deleted region (Supporting Information Table S1).

Blackgrass (*Alopecurus myosuroides* Huds.) and velvetgrass (*Holcus lanatus* L.) grains were obtained from the USDA‐ARS GRIN (Germplasm Resources Information Network) or from germplasm held at Rothamsted Research. For anatomical analysis of blackgrass, GRIN accessions PI 289645 and PI 204402, originating from Spain and Turkey respectively, were used. For VIGS in blackgrass, a line derived from the Peldon biotype was used (Mellado‐Sánchez *et al*., 2020). For velvetgrass, GRIN accessions PI 302907 and PI 311415, both from Spain, were used. Brachypodium and velvetgrass were grown under long day conditions (20 h : 4 h, light : dark), at 28°C. Blackgrass was grown under short‐day conditions (10 h : 14 h, light : dark), at 22°C except for VIGS, details for which are given below.

Awns were collected from various species as follows: wheat (*Triticum aestivum* L.) and rye (*Secale cereale* L.) growing at the UMass Crop Research Center, ‘Steptoe’ barley (*Hordeum vulgare* L.) grown in the UMass Morrill Glasshouse, and *Bromus tectorum* L. (cheatgrass) growing outside the UMass Morrill Science Center. Vouchers for *Alopecurus myosuroides* (MASS 00445199), *Bromus tectorum* (MASS 00445200), and *Holcus lanatus* (MASS 00445198) were deposited at the University of Massachusetts herbarium.

### Staining and microscopy

Awn and leaf sections were made by hand and immediately moved to sterile water on a microscope slide to keep fresh. When several sections had been collected, the water was exchanged for Toluidine Blue for 1–2 min, after which the sections were washed to remove excess stain. A coverslip was applied, and sections were imaged. For stained sections, a Zeiss Axioplan microscope with AmScope digital camera (MU633‐BI) was used.

For scanning electron microscopy, a JEOL JCM‐6000Plus was used. For developing flowers, fresh tissue was dissected immediately before imaging and mounted on a stub with oven‐bake clay (Sculpey III). For senesced awns, the whole awn was mounted in the same manner. In all cases, images were collected under high vacuum and low voltage (5 kV).

### Awn clipping

The awn clipping experiment was performed twice in the same manner, except where noted. Brachypodium grains from the Bd21‐3 inbred line were grown in 2.5 inch pots, with four germinants per pot. In each pot, plants were randomly assigned to a treatment, with one glume removal, one no treatment, and two awn removal plants. At flowering, in the awn removal treatment, awns were cut off as they appeared. In the glume removal treatment, the lower glume on each spikelet was cut off on the first day that awns were visible. Plants in the no treatment condition were not clipped, but were handled a similar amount as the other two treatments.

**Fig. 2:**
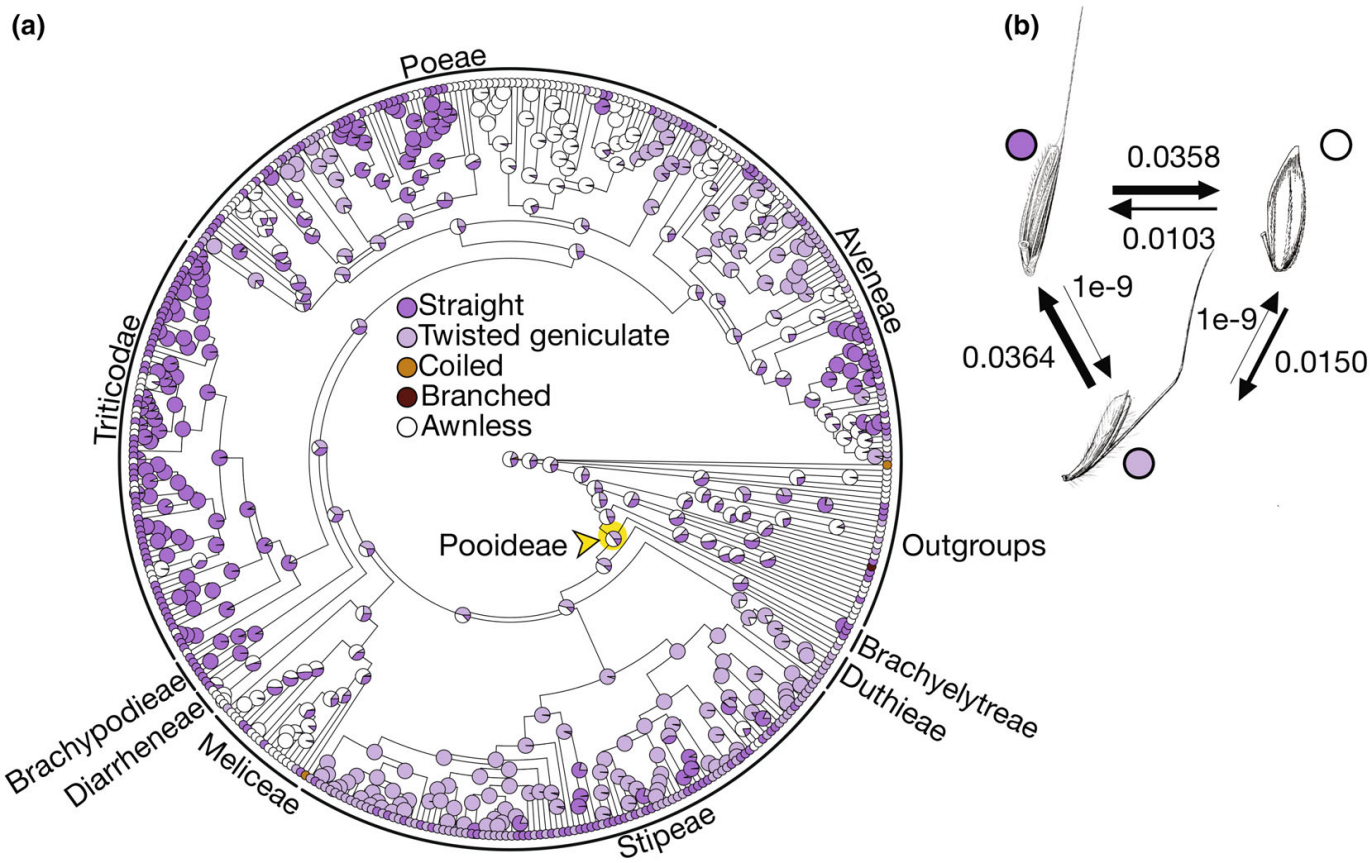
Awn morphology has a complex evolutionary history in the Pooideae (a) Ancestral state reconstruction for awn morphology. (b) Transition rates between awned and awnless states in (a). Images in (b) from Hitchcock‐Chase Collection of Grass Drawings. Yellow node indicates Pooideae most recent common ancestor.

In the second replicate experiment, at senescence, all spikelets were collected, and the number of filled grains per spikelet was counted (light blue; Fig. 2d). The first replicate was the same, but only spikelets with one or more filled grains were collected (dark blue; Fig. 2d). Filled grains were weighed. To control for differences in grain mass due to the absent awn, two different methods were used. In the first replicate (dark blue; Fig. 2c), the lemmas were fully removed from all grains before weighing. In the second replicate (light blue; Fig. 2c), the awns were cut off of the glume and no treatment lemmas before weighing. Due to their small size, grains were weighed while grouped by spikelet, and the total weight was divided by the number of grains.

### Genomic data analysis

Illumina 150‐bp paired‐end reads from *awl1* were trimmed and mapped to the *Brachypodium distachyon Bd21* genome using GSNAP (International Brachypodium Initiative, 2010; Bolger *et al*., 2014; Wu *et al*., 2016). Because *awl1* was the product of fast neutron mutagenesis, we expected that a large deletion would be the most likely mutation. Paired‐end reads that mapped uniquely but at a much greater distance than expected (‘PL’ flag in GSNAP) were visually inspected in IGV to find deletions (Thorvaldsdóttir *et al*., 2013).

### CRISPR‐Cas9 gene editing in brachypodium

Spacers targeting *BdDL* (Bradi1g69900) were designed using CRISPOR (Concordet & Haeussler, 2018). These spacers were synthesized and assembled into a guide RNA construct using the MoClo system (Miao *et al*., 2013; Engler *et al*., 2014). An additional MoClo construct was assembled containing the ubiquitin promoter from maize driving a Cas9 with introns (Grützner *et al*., 2021). These constructs were combined and Gateway cloned into a hygromycin‐containing resistance vector (O'Connor *et al*., 2014). This construct was introduced to brachypodium Bd21‐3 embryogenic callus via *Agrobacterium‐*mediated transformation using strain AGL1 (Vogel & Hill, 2008). Calli were screened with hygromycin resistance, and *Bddl‐CR* alleles were genotyped via PCR and Sanger sequencing.

### RT‐qPCR

Inflorescence meristems were collected and pooled from three wild‐type (WT) and three *awl1* plants, and immediately frozen. Total RNA was extracted using the Qiagen RNeasy Plant Kit, following the kit instructions. Five hundred nanograms of RNA was used for cDNA synthesis, using the SuperScript III RT kit (Thermo Fisher Scientific, Waltham, MA, USA). qPCR was performed with primers specific to BdDL, as well as control primers for BdUBC18 (Hong *et al*., 2008).

### VIGS in blackgrass

VIGS was performed in blackgrass following established protocols (Lee *et al*., 2015; Mellado‐Sánchez *et al*., 2020, MacGregor, 2020). Briefly, two fragments targeting regions in the gene of interest (*AmDL*, *ALOMY6G44958*) were cloned in antisense into modified barley stripe mosaic virus (BSMV) constructs following published protocols (Lee *et al*., 2015). The regions were chosen using the siRNA‐Finder (si‐Fi) software loaded with the blackgrass genome (Lück *et al*., 2019; Cai *et al*., 2023). The chosen regions had high probabilities of creating efficient siRNAs with low off‐target effects. Although the chosen regions align with six other YABBY transcription factor genes in blackgrass (*ALOMY1G03471*, *ALOMY3G12579*, *ALOMY3G15363*, *ALOMY4G27739*, *ALOMY5G34551*, and *ALOMY7G37281*), there are no stretches of pairwise identity longer than 14 nucleotides. Efficient VIGS targeting requires 21–24 nucleotides of pairwise identity (Mellado‐Sánchez *et al*., 2020). Therefore, it is unlikely that our chosen fragments would silence any annotated genes other than *AmDL*.

Two control vectors carrying the empty multiple cloning site (‘MCS’) from Mellado‐Sánchez *et al*. (2020), or a 230‐bp region from *GFP* (GenBank E17099.1) targeting from 307 to 536 from the start site were also used. A vector targeting an antisense portion of *PHYTOENE DESATURASE* (*AmPDS*) from Mellado‐Sánchez *et al*. (2020) was also used as a visual positive control to check the efficiency of VIGS (Mellado‐Sánchez *et al*., 2020). These showed clear photobleaching at 96.5% efficiency 2 wk after inoculation. Sap extracted from *N. benthamiana* leaves infected with the different BSMV constructs was used to inoculate young blackgrass seedlings 2 wk after they had been vernalized for 5 wk at 5°C. The plants were grown at 16 h : 8 h, light : dark, 27°C : 21°C for 2 wk after rub inoculation to allow the virus to establish and then moved to 16 h : 8 h, light : dark, 21°C : 15°C until flowering.

## Results

### Awns have been gained, lost, and regained many times in the Pooideae

To examine the evolutionary history of awns, and to identify cases of independent awn emergence for further study, we reconstructed the most likely ancestral awn states in the Pooideae, focusing on lemma awns. We mapped awn presence onto a time‐calibrated tree containing *c*. 10% of the species in the subfamily (Schubert *et al*., 2019; Fig. 1d). Reconstructions at deep nodes were ambiguous, with awned and awnless lemmas predicted to be equally likely in the common ancestor of all Pooideae species. However, we predicted several instances of gain and loss of awns at shallower nodes. Awns emerged roughly twice as often as they disappeared in the Pooideae (41 gains, 22 losses in our sampling; Fig. 1d,e; Tables S2, S3). Awns emerged and disappeared both within genera (27 intrageneric gains and 15 intrageneric losses), and before the divergence of larger clades (five gains and seven losses on branches leading to clades containing multiple genera). Because the phylogeny we used includes a small fraction of Pooideae species (Kellogg, 2015; Schubert *et al*., 2019), these numbers of transitions are likely underestimates. To determine whether awns were emerging and disappearing at random in the Pooideae, we estimated the phylogenetic signal of awn presence using the *D*‐statistic (Fritz & Purvis, 2010). Phylogenetic signal estimates the tendency of a trait to be similar between closely related species, rather than evolving randomly across a phylogeny (Kamilar & Cooper, 2013). The *D*‐statistic was estimated at 0.2667, indicating that awn presence was not evolving randomly (*P* = 0). Thus, awn evolution is a labile, but not random, process in the Pooideae.

### Awn morphology, anatomy, and micromorphology varies across the Pooideae

To assess how awn morphology changed across the Pooideae, we mapped awn morphology, awn length, and awn insertion point (Figs 2a, S1, S2). Most species in our dataset showed one of three morphologies: (1) no awns, (2) twisted geniculate awns, or (3) straight awns (Fig. 2a,b). Coiled or branched awns were present only in *Streblochaete longiarista* and in some outgroup species. As with awn presence, character states at deeper nodes were ambiguous, but shallower nodes within tribes showed clear patterns. For example, the Triticodae (supertribe containing Triticeae (wheat and relatives), Bromeae, and Littledaleeae; Orton *et al*., 2021) only had straight awns. By contrast, the other large tribes (Poeae, Aveneae, and Stipeae) all contained a mix of species with each awn type. The longest awns were specific to Stipeae and wheat, barley, and their close relatives (Fig. S1), reaching up to 25 times the length of the lemma body, as in *Nassella tenuissima* (Stipeae). On average, awns in this subset of the Pooideae are *c*. 3.6 times the length of the lemma, and only 31% of awned species have awns equal to or shorter than the length of their cognate lemma bodies. Awns were abaxially inserted in species from the Meliceae, Poeae, and Aveneae, as well as in a single Triticeae species (Fig. S2). Straight awns rarely shifted to twisted geniculate, but twisted geniculate often shifted to straight. Additionally, twisted geniculate awns have evolved from the awnless state about as often as straight awns (Fig. 2b).

**Fig. 3:**
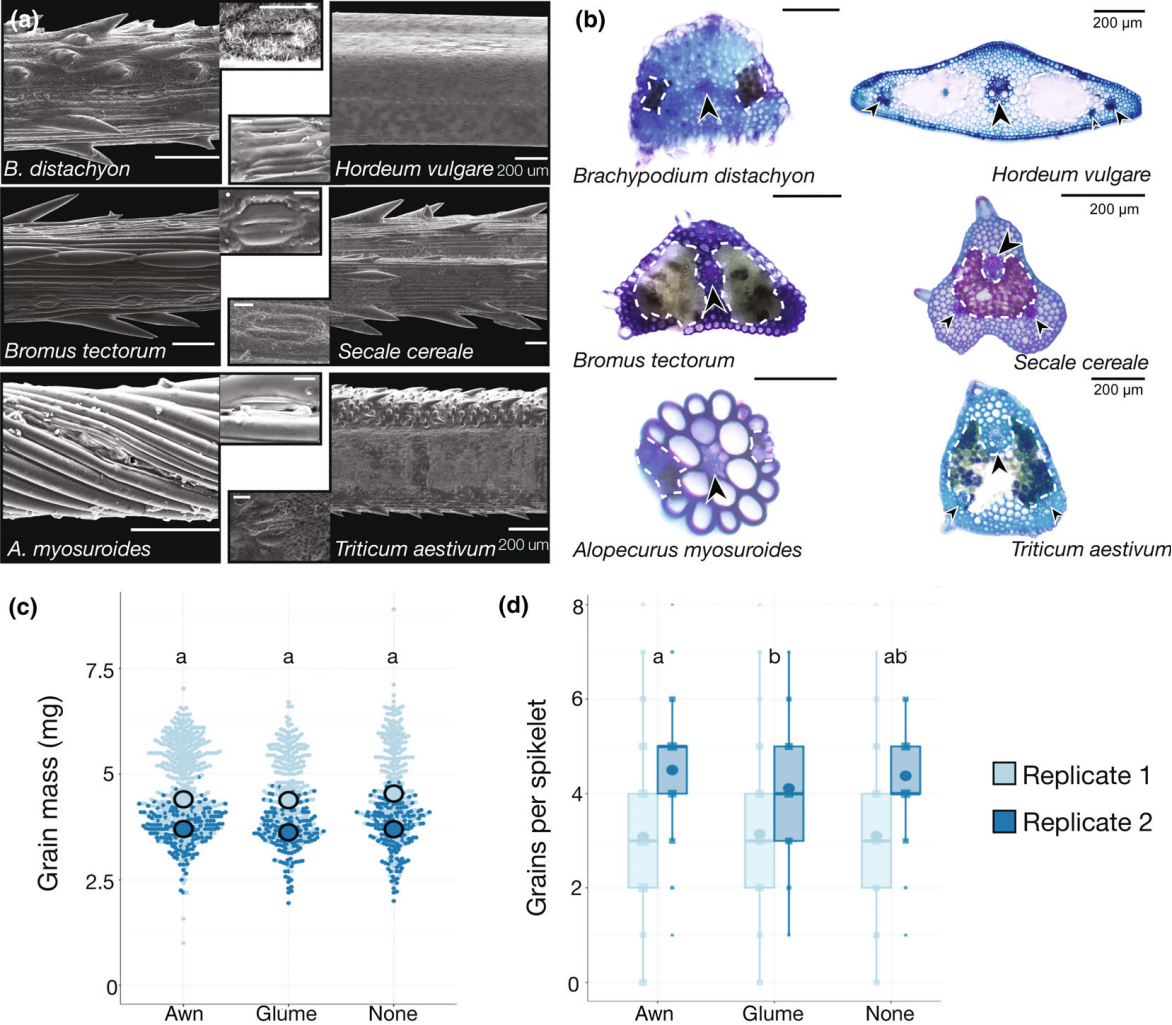
Awns in the Pooideae differ anatomically and functionally. (a) Scanning electron micrographs (SEMs) of awn sections from six Pooideae species, stomata in insets (bars, 10 μM). (b) Transverse sections of awns from the same six species, chlorenchyma (white dotted lines) and vasculature (arrows) noted. Bars, 100 μm throughout unless noted. (c) Awn removal has no effect on grain mass in brachypodium. (d) Awn removal has no effect on filled grains per spikelet in brachypodium. Light and dark blue denote data from replicate experiments. Letters indicate significantly different groups (analysis of variance, Tukey's *post hoc*). Boxplots, generated with ggplot2 in R, indicate calculated minima and maxima (whiskers), first and third quartile (box), median (thick line), and outliers (dots above or below whiskers).

We next examined awn anatomy and micromorphology in six Pooideae species representing two or three independent awn derivations (Fig. 3a,b). First, awns in *Alopecurus myosuroides* (blackgrass) were independently derived from those in the other five species we examined (Fig. 1d, node 3). Second, the Triticodae species *Triticum aestivum* (wheat), *Hordeum vulgare* (barley), *Secale cereale* (rye), and *Bromus tectorum* (cheatgrass) share a derivation (Fig. 1d, node 1). Third, awns in brachypodium may have been independently derived from those in the Triticodae (Fig. 1d, node 2). Awns in these species differed in cuticular wax formation and prickle density (Fig. 3a). In cross‐section, the straight awns all had many small cells, while the twisted geniculate awn of blackgrass was composed of few large cells, with only two cell layers surrounding the central vascular bundle (Fig. 3b). Thus, awn morphology, anatomy, and micromorphology differ across the Pooideae, suggesting that independently derived awns serve diverse functions in the Pooideae.

### Awn removal did not impact grain mass or grain number in brachypodium

We next examined whether function is conserved between the awns of brachypodium and *Triticum aestivum* (wheat) or *Hordeum vulgare* (barley). Brachypodium, barley, and wheat have straight awns with similar cell types and organization, including stomata in rows along the abaxial awn surface and chlorenchyma, suggesting photosynthetic capacity (Fig. 3a). However, the awns of brachypodium are much shorter than those of wheat and barley, and have less chlorenchyma. Brachypodium awns may have been independently derived from those in wheat and barley (Figs 3b, S1, S2). Across many independent investigations, awns in wheat and barley contribute to grain mass, likely by contributing photosynthate to developing grains (Harlan & Hulton, 1920; Miller *et al*., 1944; Grundbacher, 1963; Li *et al*., 2006, 2023; Abebe *et al*., 2009; Maydup *et al*., 2010; Liller *et al*., 2017; Swarts *et al*., 2017; Sanchez‐Bragado *et al*., 2023). To determine whether brachypodium awns similarly affected grain filling, we performed an awn removal experiment.

In two independent experimental replicates, we either (1) removed all awns, or (2) removed a single glume from developing spikelets (to control for wounding), or (3) left plants untreated. Mean grain mass was similar between control and treatment plants in both experimental replicates (Replicate 1: *P* = 0.144, Replicate 2: *P* = 0.332, ANOVA; Fig. 3c; Tables S4, S5). A power analysis revealed that a sample size of 279 (Replicate 2) or 587 (Replicate 1) grains per treatment would be sufficient to detect a 4% change in mean grain mass (Cohen's *d* = 0.29, α = 0.01, power = 80%), which is at the low end of what is seen in wheat (Sanchez‐Bragado *et al*., 2023). Our awn clipping experiments included measurements for 629–1213 grains per treatment (Table S5), well above these minima. Similarly, awn removal did not affect grain number per spikelet in either experimental replicate (Fig. 3d). Grain number per spikelet was significantly lower in plants that had their glumes removed than in plants that had their awns removed, but in only one experimental replicate (Replicate 1: *P* = 0.916, Replicate 2: *P* = 0.005, ANOVA). A power analysis estimated that a minimum of 11 (Replicate 1) or 14 (Replicate 2) spikelets per treatment would be required to detect a difference in grain number per spikelet beyond the variation seen in untreated plants (Cohen's *d* = 1.41, α = 0.01, power = 80%). Our awn clipping experiments included measurements for 153–393 spikelets per treatment (Table S5). Given that our sample numbers were well above the estimated minimum requirements, a bigger experiment would probably not reveal a function for brachypodium awns in grain filling. Brachypodium awns are shorter and narrower than those of wheat and barley (Figs 3b, S1), which may limit their potential for contributing to grain filling. However, awns in other species may make particular contributions to grain filling under drought stress (Ntakirutimana & Xie, 2020; Sanchez‐Bragado *et al*., 2023). A function for brachypodium awns in grain filling might be detected under drought conditions.

### Independently derived awns had similar developmental trajectories

If a latent leaf developmental program underlies the repeated evolution of awned lemmas, then we expect awned lemma development to follow the stereotypical grass leaf developmental pattern. In grass leaves, the blade and sheath compartments both initiate early, but the blade begins expanding first. The sheath compartment expands later, concomitantly with the ligule (Lewis & Hake, 2016; Strable & Nelissen, 2021). To evaluate whether awned lemmas followed this pattern, we tracked awn development in three species with independently derived awns (Fig. 1d) and differing awn morphologies: brachypodium (straight), *Alopecurus myosuroides* (blackgrass, abaxially inserted close to the base, and twisted geniculate), and *Holcus lanatus* (velvetgrass, abaxially inserted close to the tip, and twisted geniculate). Velvetgrass was not included in anatomical analyses due to the small diameter (< 100 μm) and length (*c*. 2 mm) of its awns, and limited availability of flowers.

**Fig. 4:**
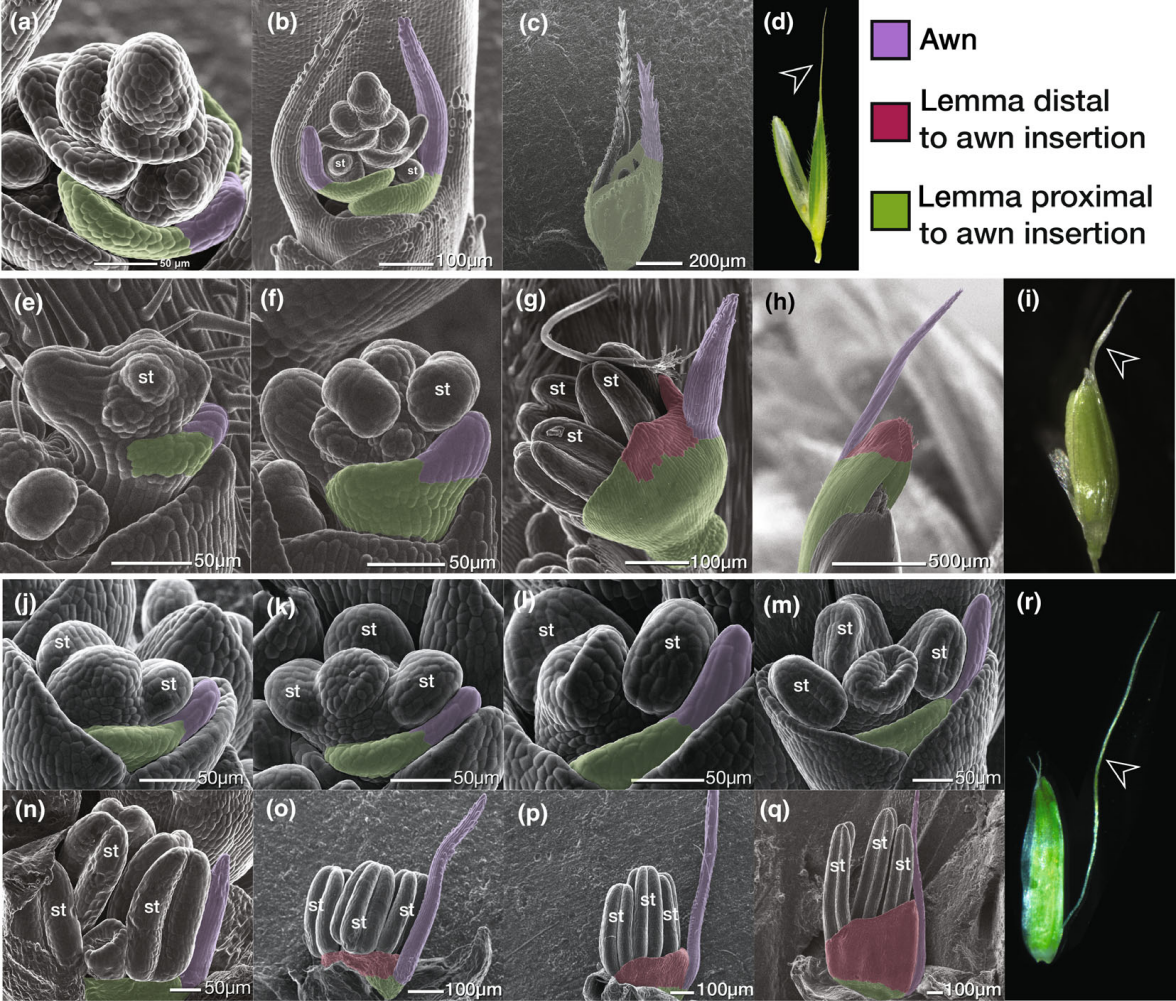
Developmental trajectory of awns is shared across three species with independently derived awns. (a–c) Scanning electron micrographs (SEMs) of awn development in brachypodium. (d) Mature brachypodium flower, awn noted with arrow. (e–h) SEMs of awn development in velvetgrass (*Holcus lanatus*). (i) Mature velvetgrass flower, awn noted with arrow. (j–q) SEMs of awn development in blackgrass (*Alopecurus myosuroides*). (r) Mature blackgrass flower, awn noted with arrow. Throughout, SEMs are false‐colored to indicate putative leaf compartment homologies. st, stamen or stamen primordium.

In brachypodium, the lemma primordium appeared very early, subtending the floral meristem, with an acute tip. This acute tip extended into an awn, with each subsequent, younger awn slightly shorter than the older one on the lemma below it (Fig. 4a,b). Awns started elongating before stamen primordia initiated, and differentiated to produce prickles from tip to base (Fig. 4b). The lemma compartment proximal to the awn insertion point extended relatively late in development, once the carpels had begun to differentiate (Fig. 4c). Mature brachypodium lemmas had no visible compartment distal to the awn insertion point (Fig. 4c,d).

In velvetgrass (*Holcus lanatus*), the lemma primordium also appeared early and had an acute tip (Fig. 4e). The velvetgrass spikelet has only one awned flower (Fig. 4i). The awned lemma expanded while the floral organs developed, with both the acute tip and lower lemma tissue expanding (Fig. 4f). Later, the awn extended and produced prickles as the stamens continued developing, and the lemma compartment distal to the awn insertion point extended (Fig. 4g). As in brachypodium, the compartment proximal to the awn insertion point formed the body of the lemma (Fig. 4h).

In blackgrass, the lemma primordium appeared early in floral development, had an acute tip, and was morphologically very similar to the awn in brachypodium and velvetgrass (Fig. 4j). However, in contrast to brachypodium and velvetgrass, lemma development paused after this initial patterning, while the inner floral organs initiated (Fig. 4k,l). Only when the anthers had begun forming locules did the awn extend (Fig. 4m,n). Once the awn extended, the lemma tissue distal to the awn insertion point appeared and extended (Fig. 4o–q). At maturity, the awn was inserted very near the base of the lemma, and the compartment below the awn insertion point extended minimally (Fig. 4r). If the awn is indeed homologous to the leaf blade and the base of the awned lemma homologous to the leaf sheath (Duval‐Jouve, 1871; Thi‐Tuyet‐Hoa, 1965), then, based on position, the compartment distal to the awn insertion point would be homologous to the ligule. In this case, most of the lemma body in blackgrass would be composed of ligule tissue; a hypothesis that needs further exploration.

Despite differences in morphology, these species share a trajectory of (1) initiation of the lemma base and awn; (2), extension and differentiation of the awn; (3) later extension of the lemma base (as in brachypodium and velvetgrass), and of the compartment distal to the awn insertion point (if present, as in blackgrass and velvetgrass). This trajectory is similar to the pattern of grass leaf blade development, where the sheath and blade initiate first, followed by blade extension and differentiation, and lastly sheath and ligule extension and differentiation (Lewis & Hake, 2016; Strable & Nelissen, 2021). In addition, awned lemmas in all three examined species were initially very similar (Fig. 4a,e,j), but diverged in form over the course of development. The developmental trajectory and early patterning shared between independently derived awns, with differing adult morphologies, suggests a similar developmental program acting in all three species.

### *DROOPING LEAF* regulates development of independently derived awns with differing morphologies

If developmental conservation underlies the replicated evolution of awns, then we expect conserved genetic pathways to regulate the independent derivations of awns. To evaluate this prediction, we explored the function of the *YABBY* transcription factor gene *DROOPING LEAF (DL)*, which regulates awn development in rice and barley (Toriba & Hirano, 2014; Zhang *et al*., 2024), and carpel and leaf blade midrib development in rice and maize (Ohmori *et al*., 2011; Toriba & Hirano, 2014; Strable *et al*., 2017; Strable & Vollbrecht, 2019).

**Fig. 5:**
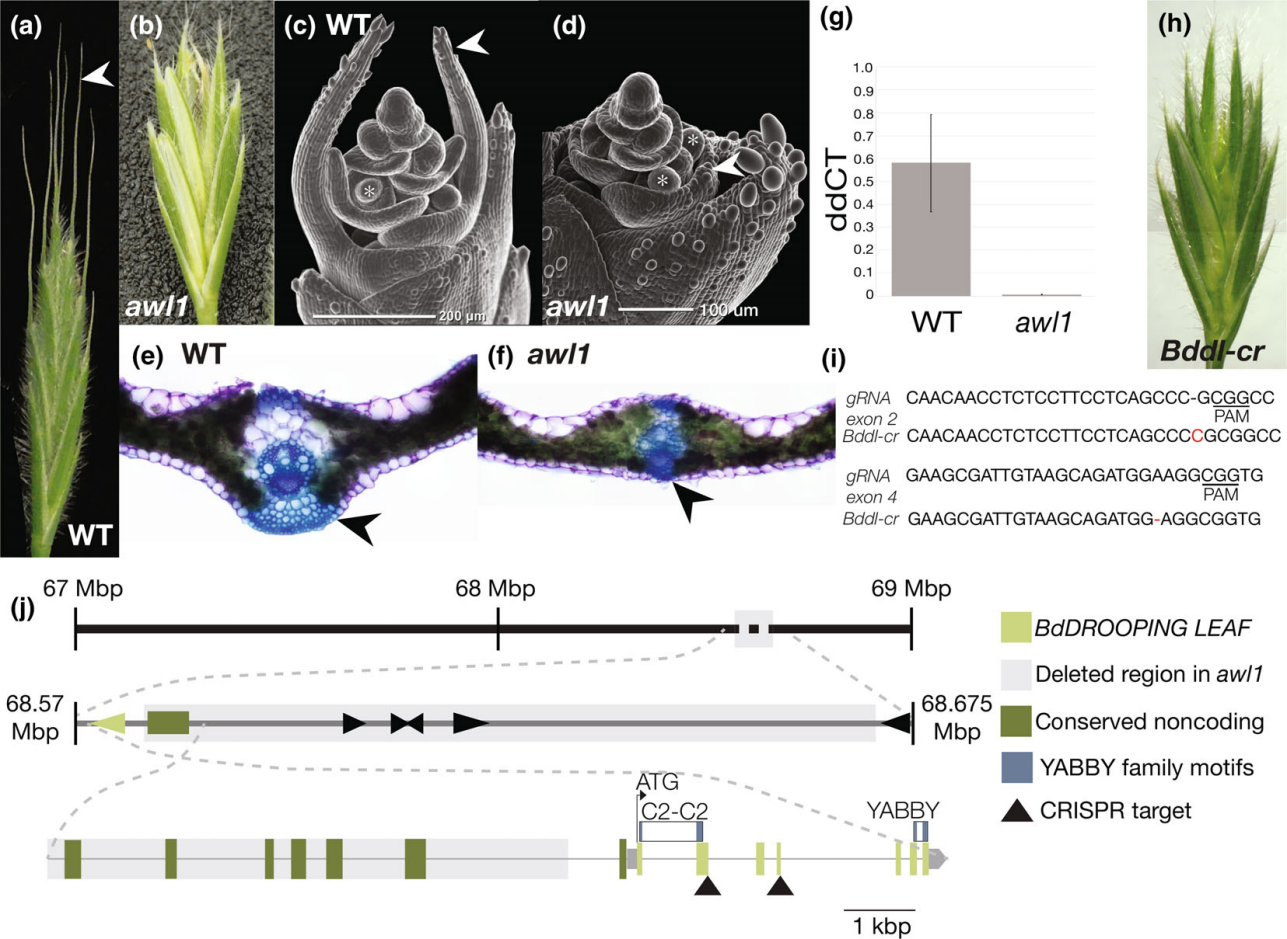
*BdDROOPING LEAF (BdDL)* is necessary for awn development in brachypodium. (a, b) Spikelet phenotypes (wild‐type (WT), *awl1* mutant), awns noted with arrow. (c, d) The *awl1* mutant does not initiate awn growth. Stamen primordia of similar age marked with asterisk. (e, f) The *awl1* mutant lacks leaf midribs (arrow). (g) The *awl1* mutant has very low expression of *BdDL* in floral tissue. Error bars represent SD. (h) The *Bddl‐cr* mutant phenocopies *awl1*, lacking awns. (i) Edited sequence in *Bddl‐cr*, biallelic single nucleotide mutations in exons 2 and 4. (j) Genomic region of chromosome 1 in *awl1* mutants, with deletion, *BdDL*, other genes, upstream conserved noncoding sequence, and CRISPR construct targets shown.

We first mapped and cloned an existing brachypodium mutant, *awnless1 (awl1*) that phenocopies other *dl* mutants (Yamaguchi *et al*., 2004; Derbyshire & Byrne, 2013; Strable & Vollbrecht, 2019). The *awl1* mutant is awnless and lacks leaf midribs (Fig. 5a–f). Whole‐genome sequencing of *awl1* mutants revealed a deletion that co‐segregates with *awl1* phenotypes (*P* = 0.718, chi‐squared test; Figs 5g, S3). This deletion removes four genes, and a stretch of conserved noncoding sequences upstream of *BdDL*. Importantly, *BdDL* expression was undetectable in *awl1* mutants using reverse transcription quantitative polymerase chain reaction (Fig. 5h). We generated a *Bddl* mutant using CRISPR‐Cas9 genome editing, which recapitulated the awnless phenotype of *awl1* (Fig. 5i). This mutant was biallelic, with single base pair edits in exons 2 and 4 predicted to result in premature stop codons (Fig. 5j). Thus, *BdDL* is required for awn initiation in brachypodium. Reduced expression of *Bddl*, associated with deletion of *BdDL* regulatory regions, likely underlies the awnless phenotype of *awl1* mutants.

We next tested whether *DL* is necessary for awn development in blackgrass using virus‐induced gene silencing (VIGS). The twisted geniculate awns in blackgrass were derived independently from the straight awns of brachypodium and rice (Fig. 1d). We used VIGS (Mellado‐Sánchez *et al*., 2020) to knock down *AmDL* (ALOMY6G44958) expression in blackgrass. Blackgrass has a single *DL* homolog, reducing the likelihood of off‐target effects (Cai *et al*., 2023). We designed three constructs targeting different regions of the *AmDL* mRNA, and construct containing either 230 bp of *GFP* (GenBank: E17099.1) or an empty multiple cloning site (MCS) as negative controls for off‐target effects. Although there are short stretches of identity between our VIGS constructs and six other *YABBY* genes in the blackgrass genome, none of these stretches are long enough to trigger silencing (Mellado‐Sánchez *et al*., 2020; Cai *et al*., 2023). Therefore, we think off‐target silencing is unlikely.

**Fig. 6:**
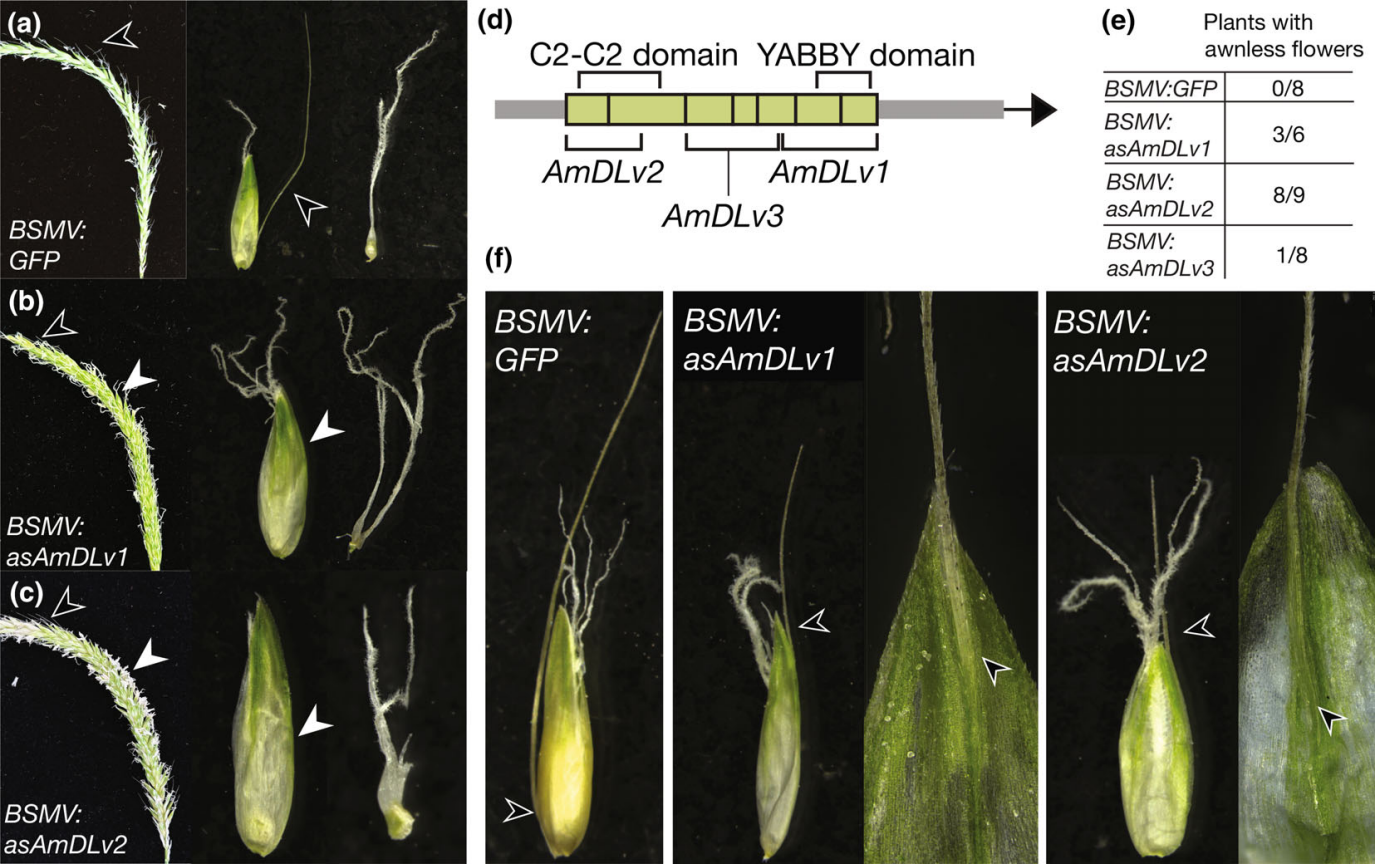
*AmDROOPING LEAF (AmDL)* is necessary for awn development in blackgrass (*Alopecurus myosuroides*). (a–c) Phenotypes of plants treated with the control BSMV:GFP (a) construct or with a construct targeting *AmDL* (b, c). Whole spike, single floret, and carpel shown for each. (d) Diagram of *AmDL* with VIGS target regions and conserved domains noted. (e) Number of plants treated and with awnless phenotype for each construct. (f) Changes in insertion point observed in both *AmDL* VIGS treatments, compared with the WT insertion point as seen in the BSMV : GFP control. Awns noted with black arrows, lack of awns noted with white arrows.

Targeting *AmDL* with VIGS resulted in differences in awn initiation, length, and placement. Despite differences in efficiency, all three constructs targeting *AmDL* resulted in awnless lemmas (12/23 plants, total of 15 spikes, Fig. 6e). The two constructs with the strongest response also resulted in aberrant carpels, as in *dl* mutants in rice and maize (Nagasawa *et al*., 2003; Strable & Vollbrecht, 2019). All plants treated with the *GFP* or MCS (empty vector) negative control constructs had normal awn development (24/24 plants; Figs 6a–c, S4).

Blackgrass awns are normally inserted near lemma bases. Interestingly, in more than half (8/15) of the *AmDL‐VIGS* spikes with awnless flowers, other flowers on the affected spike had shorter awns. In these flowers, the awn was inserted near the center of the lemma and partially fused to the lemma, rather than free as in untreated and control blackgrass plants (Fig. 6e). This change in insertion point suggests that *DL* has a role in partitioning the leaf primordium along the proximal–distal axis. This could provide a mechanism for modulating awn insertion point, an important taxonomic trait (Kellogg, 2015; Peterson *et al*., 2019). Together, these results show that *DL* is necessary for awn development in at least three independent derivations of awns (Fig. 1d) – rice (Toriba & Hirano, 2014), brachypodium (Fig. 5b), and blackgrass (Fig. 6b,c). A *DL* homolog also regulates awn development in barley (Zhang *et al*., 2024), which may represent a fourth independent derivation of awns (ancestral state estimations equivocal; Fig. 1d). Thus, although awns in these species are independently derived and differ in form and function, *DL* is a key regulator of awn development in all cases.

## Discussion

Despite a complex evolutionary history with many independent derivations, awns are likely homologous to leaf blades, and share developmental patterns and pathways. Awns evolved independently many times in the Pooideae (Fig. 1d; Humphreys *et al*., 2011; Teisher *et al*., 2017; Zhang *et al*., 2022; Petersen & Kellogg, 2022). Vascular anatomy was similar between brachypodium, wheat, and barley awns (Fig. 3b), but awn function may not be conserved. Awn removal in brachypodium had no effect on measured grain traits in our experimental conditions (Fig. 3c). Independently derived awns, with differing morphologies and hypothesized functions, had developmental trajectories similar to each other and to grass leaves (Fig. 4; Lewis & Hake, 2016; Strable & Nelissen, 2021). Furthermore, *DL* was necessary for awn initiation in both brachypodium and blackgrass, and may have a role in regulating awn insertion point (Figs 5, 6). Together, these data matched our predictions that despite divergent form and function, independently derived awns have (1) conserved developmental trajectories and (2) conserved genes regulating their development. Thus, a conserved leaf blade developmental program may underlie the replicated evolution of awns.

Many examples of replicated evolution are adaptations to particular environmental conditions: root loss in aquatic Lemnaceae, xerophytic forms in *Euphorbia*, eye loss in cavefish, benthic phenotypes in sticklebacks, and mandible shape in beetles (Horn *et al*., 2012; McGee & Wainwright, 2013; Baulechner *et al*., 2020; Sifuentes‐Romero *et al*., 2020; Ware *et al*., 2023). For example, in beetles, mandible shape is strongly correlated with diet, regardless of phylogenetic history (Baulechner *et al*., 2020). While awns are likely homologous, they differ morphologically and functionally, and many awns may lack specific or adaptive functions. Indeed, the replicated emergence of awns need not always be adaptive (Gould & Lewontin, 1979; Gould, 2002). Instead, awns may arise as frequently as they do as a consequence of development, and provide phenotypic variation for natural selection to act on in some circumstances.

Diversity in awned lemma morphology may be driven, in part, by differential growth of lemma compartments. Awns themselves are much narrower than vegetative leaves, suggesting that lateral and marginal leaf compartments are suppressed or reduced in awned lemmas (Richardson *et al*., 2021; Satterlee *et al*., 2023), or some other mechanism leads to mediolaterally constricted awns. In brachypodium, no compartment distal to the awn insertion point was visible, leading to a long apically inserted awn (Fig. 4d). In velvetgrass, there was a small compartment distal to the awn insertion point, leading to a sub‐apically inserted awn (Fig. 4i). In blackgrass, the compartment above the awn insertion point expanded far more than the base of the lemma, leading to a long, abaxially inserted awn (Fig. 4r). If the compartment distal to the awn insertion point is homologous to the ligule, as is suggested by its position (Figs 1, 4), then most of the lemma body in blackgrass is ligule tissue, although this hypothesis requires further testing. Regardless of homologies, differential expansion and differentiation of lemma compartments was an important factor driving morphological differences between awned lemmas in these three species. This is similar to differential growth in leaves contributing to morphological diversity (Kaplan, 2001), or in heterostylous *Primula* flowers (Webster & Gilmartin, 2006). Thus, differential growth downstream of deeply conserved organ patterning processes can contribute to diversity in form at adulthood.

Many aerial organs, including lemmas and floral organs such as carpels, are modified leaves (Von Goethe & Miller, 2009). In addition to its role in leaf and awn development, *DL* also regulates carpel development (Toriba & Hirano, 2014; Strable & Vollbrecht, 2019). The brachypodium *awl1* mutant, blackgrass treated with *BSMV:asAmDL* (Figs 5, 6), maize mutants *Zmdrl1* and *Zmdrl2*, and rice *dl* mutants all have aberrant carpel development (Ohmori *et al*., 2011; Toriba & Hirano, 2014; Strable *et al*., 2017; Strable & Vollbrecht, 2019). Similarly, the *Arabidopsis thaliana DL* ortholog *CRABS CLAW* (*CRC*) regulates nectary and carpel development (Lee *et al*., 2005), although *CRC* may have an ancestral role in leaf development (Fourquin *et al*., 2014). *DL* is not alone in having dual roles in awns and carpels: a *SHORT INTERNODES/STYLISH (SHI)* homolog has a role in both awn and carpel development in barley (Yuo *et al*., 2012). In rice, an epidermal patterning factor‐like (EPFL) protein regulates both awn length and grain length, which is likely associated with carpel size (Jin *et al*., 2016). Indeed, a growing number of genes are implicated in both carpel and lemma development (Zhang *et al*., 2016; Ma *et al*., 2019; Shoesmith *et al*., 2021; Jing *et al*., 2023). In some of these cases, carpel development is limited by physical constraint of the developing grain due to decreased lemma size (Ren *et al*., 2018; Zhang *et al*., 2024). In those cases where physical constraint is not a factor, we suggest that dual roles for genes in lemma and carpel development are due to their shared evolutionary origins (Von Goethe & Miller, 2009). However, all floral organs are leaf homologs, and paleas and lodicules are not strongly affected in *dl* mutants. Mutations in *DL* orthologs have their strongest effects on medial and distal leaf homolog compartments (Nagasawa *et al*., 2003; Yamaguchi *et al*., 2004; Von Goethe & Miller, 2009; Ohmori *et al*., 2011; Toriba & Hirano, 2014; Strable *et al*., 2017; Strable & Vollbrecht, 2019). Perhaps paleas and lodicules lack these compartments, similar to awnless lemmas, and are thus only weakly affected.

*DL* expression is unlikely to be a singular ‘on/off’ switch in awn evolution for a few reasons. First, *DL* orthologs have only a subtle role in proximal–distal patterning in rice, brachypodium, and maize (Yamaguchi *et al*., 2004; Strable *et al*., 2017). Second, *DL* expression is not significantly different between awned vs awnless rice (Toriba & Hirano, 2014), suggesting that processes downstream of *DL*, rather than *DL* itself, drive differential awn development in rice. However, at deeper phylogenetic levels, there are differences in *DL* expression. In maize, which is awnless, *DL* orthologs are expressed in lemma primordia only very early on in development (Strable & Vollbrecht, 2019). In rice, sorghum, and wheat, which can all be awned, *DL* expression in the lemma midrib persists until later on in development (Ishikawa *et al*., 2009; Toriba & Hirano, 2014). This is similar to *DL* expression in vegetative leaves of maize, sorghum, rice, and wheat, where *DL* is expressed throughout very early leaf primordia, but later becomes restricted to the midrib region (Yamaguchi *et al*., 2004; Ishikawa *et al*., 2009; Strable *et al*., 2017). Thus, variation in *DL* expression timing may contribute to variation between genera. However, leaf developmental networks are complex (Conklin *et al*., 2019), as is awn evolutionary history (Petersen & Kellogg, 2022). The mechanisms underlying awn emergence and disappearance likely differ between species.

Developmental constraints are often considered only in a restrictive sense, preventing phenotypes from being accessed. We highlight the positive ability of developmental constraints to facilitate replicated evolution. Our results highlight the fundamental relatedness of genetic processes in plants, and suggest that positive developmental constraint may be prevalent in instances of replicated evolution in plants, where many aerial organs are homologous to leaves (Von Goethe & Miller, 2009).

## Competing interests

None declared.

## Author contributions

EP, DRM and MEB designed the research. EP, DRM, MMH, JG, DO'C and BN performed experiments. EP analyzed data. EP and MEB wrote the manuscript. All authors read and approved the final manuscript.

## Supporting information


**Fig. S1** Ancestral state reconstruction for awn length in the Pooideae.
**Fig. S2** Ancestral state reconstruction for awn insertion point in the Pooideae.
**Fig. S3** Genotyping for the *awl1* locus.
**Fig. S4** Quantification and additional control plant images for VIGS in blackgrass.
**Table S1** Primers used in this study.

**Table S2** Data from GrassBase used in ancestral state reconstructions.
**Table S3** Summary of predicted transitions in awn presence from ancestral state reconstruction.
**Table S4** Data from both awn removal experiments.
**Table S5** Data summary from awn removal experiments.
Please note: Wiley is not responsible for the content or functionality of any Supporting Information supplied by the authors. Any queries (other than missing material) should be directed to the *New Phytologist* Central Office.

## Data Availability

Short read sequencing data are available from the NCBI short read archive (PRJNA1074741).
