# Supplementary material for: Developmental constraint underlies the replicated evolution of grass awns
Source: New Phytol. 2024 Nov 18;245(2):835–48. doi: 10.1111/nph.20268 (PMC11655442; doi:10.1111/nph.20268)
Supplement: Supplementary file 1 — Fig. S1 Ancestral state reconstruction for awn length in the Pooideae. Fig. S2 Ancestral state reconstruction for awn insertion point in the Pooideae. Fig. S3 Genotyping for the awl1 locus. Fig. S4 Quantification and additional control plant images for VIGS in blackgrass. Table S1 Primers used in this study. [file NPH-245-835-s001.pdf]

***New Phytologist* Supporting Information**

Article title: Developmental constraint underlies the replicated evolution of grass awns

Authors: Erin Patterson, Dana MacGregor, Michelle Heeney, Joseph Gallagher, Devin O'Connor, Benedikt Nuesslein, and Madelaine Bartlett

Article acceptance date: 17 October 2024

This file includes Figures S1 – S3, Table S1, and Table S5. Tables S2 – S4 are in a separate spreadsheet.

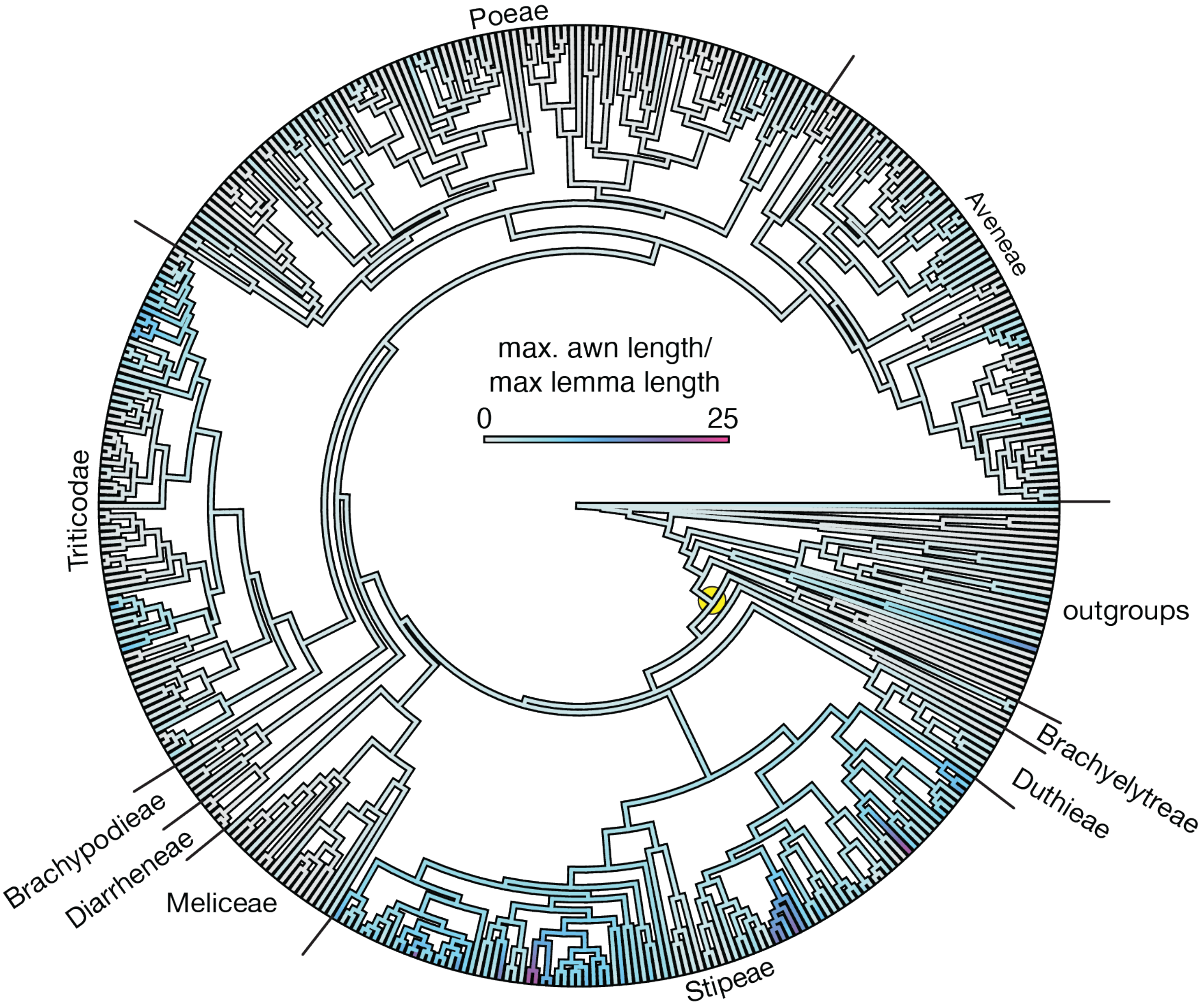

Fig. S1. Long awns have evolved multiple times in the Pooideae. Ancestral state reconstruction of maximum awn length/maximum lemma length. Yellow node: most recent common ancestor of Pooideae.

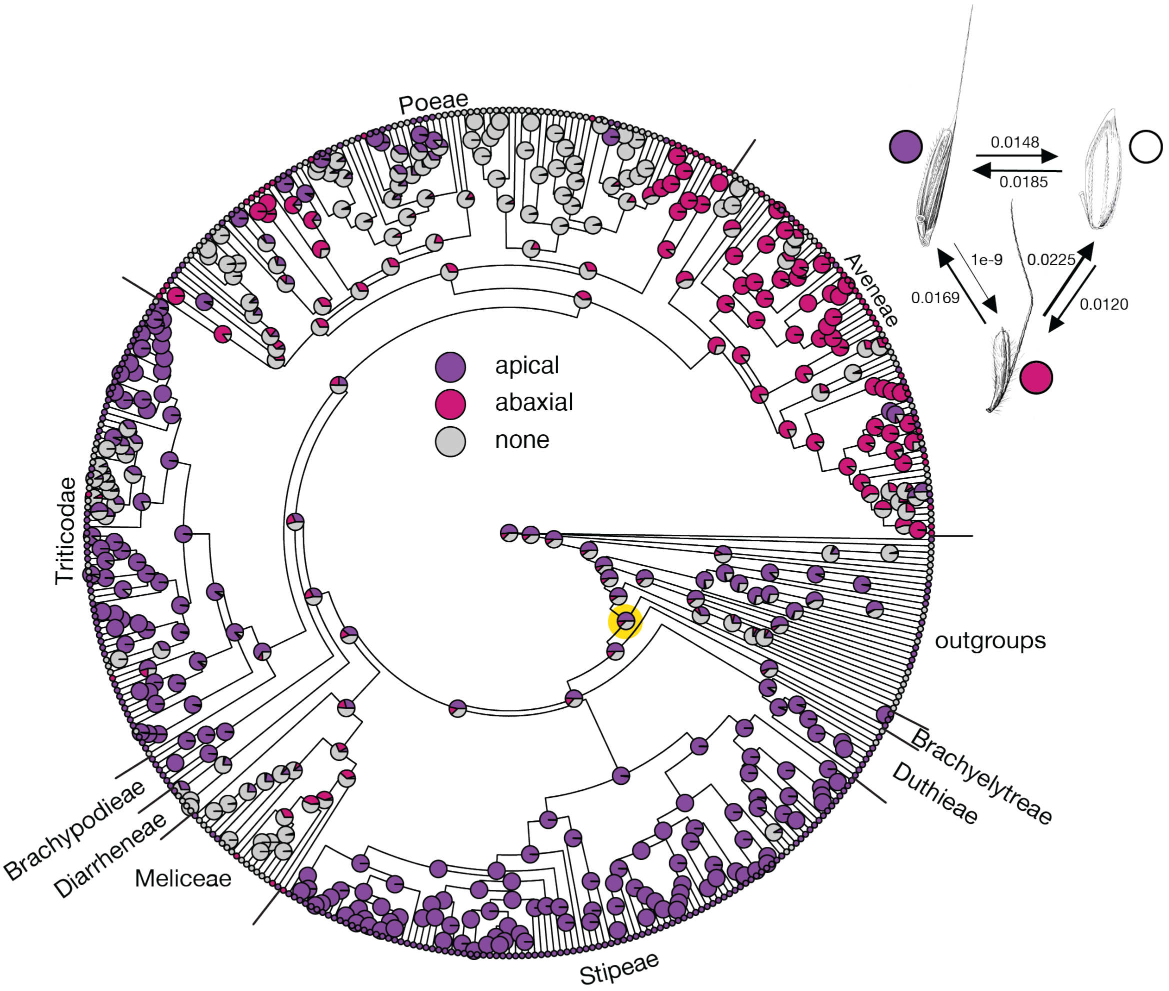

Fig. S2. Awn insertion point varies across the Pooideae. Yellow node: most recent common ancestor of Pooideae. Top right: estimated transition rates between states.

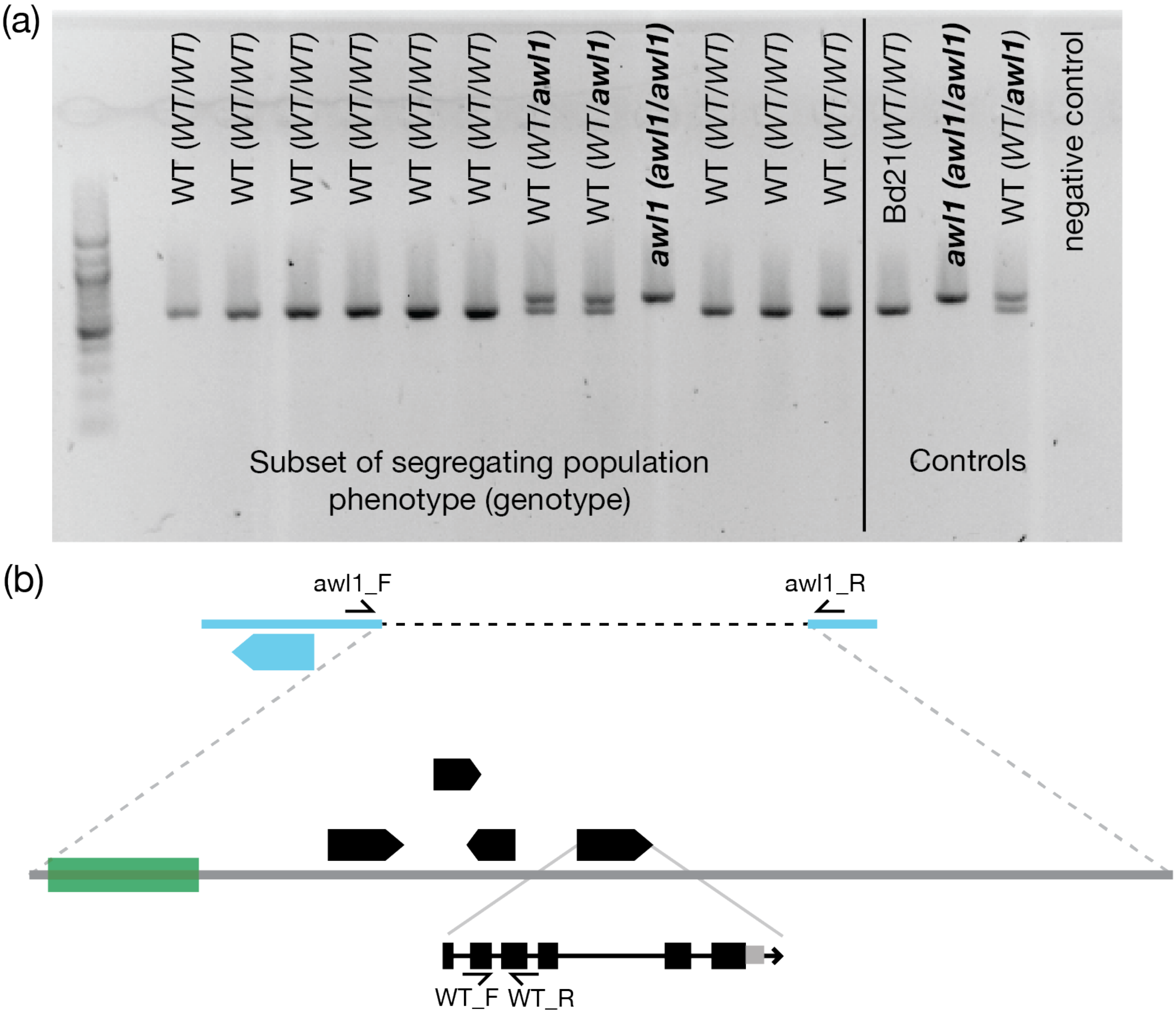

Fig. S3. *awl1* segregates as a single locus. (a) Gel electrophoresis of PCR assay for *awl1* deletion. (b) Diagram of primers for *awl1* deletion assay with multiplexed primers. Primers on either end of deletion (unable to amplify in WT), and primers within gene in deleted region (unable to amplify from *awl1*).

(a)

	plants with awnless flowers
BSMV:GFP	0/8
BSMV: MCS	0/8
BSMV: PDS	0/8

(b)

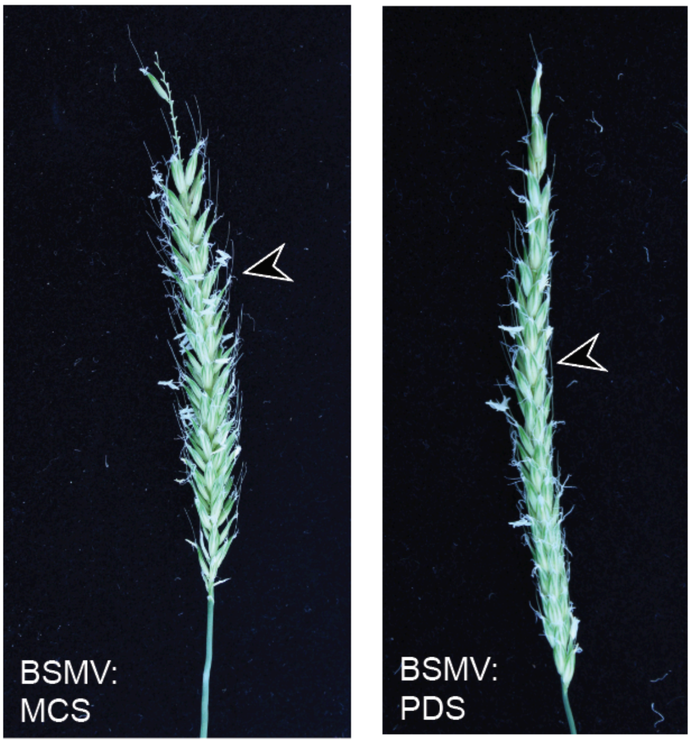

Fig. S4. Phenotypes for Multiple Cloning Site (empty vector) and phytoene desaturase (PDS) negative controls in VIGS experiment. (a) No control plants produced any awnless florets. (b) Typical spikes from the MCS and PDS controls, arrows note awns.

Table S1. Primers used in this study.

Target	Oligo Type	Sequence	Application
**BdDL FW**	qPCR Primer	TTCTTCTCCGGGGGCTTCA	Gene expression (DL)
**BdDL RV**	qPCR Primer	CGTTCCAGGGATGCACTGA	
**BdUBC18 FW**	qPCR Primer	GGAGGCACCTCAGGTCATTT	Gene expression (control)
**BdUBC18 RV**	qPCR Primer	ATAGCGGTCATTGTCTTGCG	
**BdDL-CR2 FW**	PCR Primer	GCCACGCAACACAAAGGAAT	CRISPR-Cas9 editing genotyping (exon 2)
**BdDL-CR2 RV**	PCR Primer	CTCCGGGGCTACAACCAAAT	
**BdDL-CR4 FW**	PCR Primer	GCTCCCTTTGTTGTGAAGCG	CRISPR-Cas9 editing genotyping (exon 4)
**BdDL-CR4 RV**	PCR Primer	GGCTAAGTTGTGACGAGGGA	
***awl1* deletion border FW**	PCR Primer	CACCTTTTGAAGCCCCATGT	*awl1* genotyping for gel assay in multiplex
***awl1* deletion border RV**	PCR Primer	ACCTTGTGGACACCGGCTT	
***awl1* deleted region FW**	PCR Primer	GTGTAACTGTGCAAACGAGC	*awl1* genotyping for gel assay in multiplex
***awl1* deleted region RV**	PCR Primer	ATCTCTATCCCCGTCCTTTG	
**BdDL-CR exon 2**	CRISPR-Cas9 spacer	ACCTCTCCTTCCTCAGCCCG	CRISPR guides (DL)
**BdDL-CR exon 4**	CRISPR-Cas9 spacer	GATTGTAAGCAGATGGAAGG	

Table S5. Awn removal in *Brachypodium distachyon* did not impact grain mass or grain number.

experimental replicate	experimental treatment	n (plants)	n (spikelets)	n (grains)	grain mass (mg)	grains per spikelet
1	awn removed	23	393	1213	4.90	3.44
1	glume removed	15	237	749	4.83	3.47
1	none	15	223	693	5.01	3.43
2	awn removed	22	170	765	3.70	4.50
2	glume removed	22	153	629	3.62	4.11
2	none	22	159	696	3.70	4.38
